# Supplementary material for: Content-rich biological network constructed by mining PubMed abstracts
Source: BMC Bioinformatics. 2004 Oct 8;5:147. doi: 10.1186/1471-2105-5-147 (PMC528731; doi:10.1186/1471-2105-5-147)
Supplement: Additional File 2 — The original results of the above study (non-essential files are deleted to keep the file size under the limit set by BMC bioinformatics). [file 1471-2105-5-147-S2.bz2 › chilibotAdditionalFile2/dip50/355ID8120391E4997/html/IL10_IL10RA.html]

 


 **IL10** and **IL10RA** 
  
Found 91 abstracts in PubMed, retrieved 50.  
 

 What does Google say? 
 PDF only 
| .edu only 

---

**Interactive relationship** (e.g. stimulation, inhibition, etc)

**Stimulatory relationship**- The resistance of Mphis from injured mice to IL 10  [ **IL10** ]  mediated suppression correlated with decreased IL 10  [ **IL10** ]  receptor IL 10R  [ **IL10RA** ]  expression and increased CD11b expression.  Ref: 11546654 Am J Physiol Cell Physiol, 2001
- DC maturation and IL 10  [ **IL10** ]  neutralization were associated with enhanced accumulation of the IL 10  [ **IL10** ]  receptor binding chain IL 10R1  [ **IL10RA** ]  mRNA and intracellular IL 10R1  [ **IL10RA** ]  protein.  Ref: 11254683 J Immunol, 2001
- The production of IL 10  [ **IL10** ]  by THP 1 cells stimulated with L OspA was autoregulated by a negative feedback mechanism involving the IL 10  [ **IL10** ]  receptor IL 10R  [ **IL10RA** ] .  Ref: 11895951 Infect Immun, 2002
- The acute death of trypanosome infected mice treated with anti IL 10R  [ **IL10RA** ]  antibodies in vivo was associated with focal liver necrosis, with significantly increased plasma levels of IL 6, IL 10  [ **IL10** ] , IL 12p40 and IFN gamma and enhanced synthesis of IL 6, IL 12p40 and IFN gamma by spleen cell cultures.  Ref: 12594839 Eur J Immunol, 2003
**Neutral relationship**- Only cells expressing IL 10R1  [ **IL10RA** ]  bind human IL 10  [ **IL10** ]  or vIL 10.  Ref: 11739506 J Immunol, 2001
- Crystal structure of human cytomegalovirus IL 10  [ **IL10** ]  bound to soluble human IL 10R1  [ **IL10RA** ] .  Ref: 12093920 Proc Natl Acad Sci U S A, 2002
- CONCLUSION Human chondrocytes synthesise IL 10  [ **IL10** ]  and express on their surface IL 10R  [ **IL10RA** ] .  Ref: 11332442 Clin Exp Rheumatol< MedlineTA>Clin Exp Rheumatol, 2002
- Differential IL 10R1  [ **IL10RA** ]  expression plays a critical role in IL 10  [ **IL10** ]  mediated immune regulation.  Ref: 11739506 J Immunol, 2001
- Here we report the 2.9 A crystal structure of IL 10  [ **IL10** ]  bound to a soluble form of IL 10R1  [ **IL10RA** ]  sIL 10R1.  Ref: 11485736 Immunity, 2001
- Cell surface IL 10R  [ **IL10RA** ]  was detected by incubation with biotinylated recombinant human IL 10  [ **IL10** ] .  Ref: 11332442 Clin Exp Rheumatol< MedlineTA>Clin Exp Rheumatol, 2001
- IL 10  [ **IL10** ]  knockout and normal mice treated prophylactically with anti IL 10R  [ **IL10RA** ]  demonstrated accelerated granuloma assembly and rapid parasite killing without untoward tissue inflammation.  Ref: 12379707 Infect Immun, 2002
- Up regulation of IL 10R1  [ **IL10RA** ]  expression is required to render human neutrophils fully responsive to IL 10  [ **IL10** ] .  Ref: 11490020 J Immunol, 2001
- IL 22 and IL 10  [ **IL10** ]  require different ligand specific receptor chains IL 22R and IL 10R1  [ **IL10RA** ]  but share a second receptor chain IL 10R2 to initiate cellular responses.  Ref: 12513909 J Interferon Cytokine Res, 2002
- IL 10  [ **IL10** ]  signaling via IL 10E1 is dependent on tyrosine phosphorylation in the IL 10R  [ **IL10RA** ]  alpha chain in human primary prostate cancer cell lines.  Ref: 12802285 Oncogene, 2003
- The data suggests that formation of the active IL 10  [ **IL10** ]  signaling complex occurs by a novel molecular recognition paradigm where IL 10R1  [ **IL10RA** ]  and IL 10R2 both recognize the same binding site on IL 10  [ **IL10** ] .  Ref: 11485736 Immunity, 2001
- This latter response to IL 10  [ **IL10** ]  was strictly dependent on the levels of expression of IL 10R1  [ **IL10RA** ] , which were in fact significantly increased by IL 4 in cultured neutrophils via a signaling pathway sensitive to the serine threonine kinase inhibitor H 7.  Ref: 12356680 Int Immunol, 2002
- Human IL 10  [ **IL10** ]  hIL 10 modulates critical immune and inflammatory responses by way of interactions with its high IL 10R1  [ **IL10RA** ]  and low affinity IL 10R2 cell surface receptors.  Ref: 12093920 Proc Natl Acad Sci U S A, 2002
- Cycloheximide had no effect on TNF induced IL 10  [ **IL10** ]  binding, strongly suggesting the release of a pre existing pool of IL 10R  [ **IL10RA** ]  rather than de novo receptor synthesis by PMN.  Ref: 11290804 J Immunol, 2001
- IL 10  [ **IL10** ]  is a dimeric cytokine that must engage its high affinity cell surface receptor, IL 10R1  [ **IL10RA** ] , to induce multiple cellular activities.  Ref: 12121653 Structure Camb , 2002
- Human cytomegalovirus exploits the IL 10  [ **IL10** ]  signaling pathway by expressing a functional viral IL 10  [ **IL10** ]  homolog cmvIL 10, which shares only 27% sequence identity with hIL 10 yet signals through IL 10R1  [ **IL10RA** ]  and IL 10R2.  Ref: 12093920 Proc Natl Acad Sci U S A, 2002
- These activities are dependent on the interaction of IL 10  [ **IL10** ]  with its high affinity receptor IL 10R1  [ **IL10RA** ] .  Ref: 11485736 Immunity, 2001
- In contrast to the high expression of receptors for IL 10  [ **IL10** ]  homologs in different tissues and cell lines, monocytes and NK, B, and T cells showed clear expression only of IL 10R1  [ **IL10RA** ] , IL 10R2, and IL 20R2.  Ref: 12023331 J Immunol, 2002
- IL 10  [ **IL10** ]  gene expression was detected in 10 22.7% of 44 xenografts 0 11 adenocarcinomas, 6 19 squamous cell carcinomas, 4 14 large cell carcinomas and IL 10R  [ **IL10RA** ]  expression in 8 18.2% xenografts.  Ref: 11351253 Int J Oncol, 2001
- We aimed to clone and sequence partial cDNA for rat IL 10R  [ **IL10RA** ]  and CRF2 4, determine their expression in activated rat HSC in vivo and in vitro, and examine the biological responsiveness of HSC to exogenous IL 10  [ **IL10** ] .  Ref: 12016123 Am J Physiol Gastrointest Liver Physiol, 2002
- IL 22 is an IL 10  [ **IL10** ]  homologue that binds to and signals via the class II cytokine receptor R heterodimer IL 22RA1 CFR2 4 IL 10R2, the latter chain being part of the IL 10R  [ **IL10RA** ]  complex.  Ref: 12407026 Int Immunol, 2002
- Cell proliferation and IL 10  [ **IL10** ]  production were compared in vitro, and tumour growth, percentages of necrotic areas, tumour cells positive for proliferating cell nuclear antigen PCNA, IL 10  [ **IL10** ]  receptor IL 10R  [ **IL10RA** ]  and major histocompatibility complex type I MHC I and II MHC II, as well as infiltration of macrophages, CD4 and CD8 lymphocytes and blood vessels were compared in vivo among IL 10  [ **IL10** ]  transfected and non transfected tumours.  Ref: 11872099 Immunology, 2002
- The anti apoptotic activity of IL 10  [ **IL10** ]  was significantly inhibited with a neutralizing IL 10R  [ **IL10RA** ]  Ab.  Ref: 11591769 J Immunol, 2001
- The ability of IL 10  [ **IL10** ]  to activate Stat3 tyrosine phosphorylation and SOCS 3 synthesis and to regulate IL 1 receptor antagonist and macrophage inflammatory protein 1beta release in LPS treated neutrophils correlated with this increased IL 10R1  [ **IL10RA** ]  expression, and was abolished by neutralizing anti IL 10R1  [ **IL10RA** ]  and anti IL 10R2 Abs.  Ref: 11490020 J Immunol, 2001
- Furthermore, the kinetics of TNF induced up regulation of IL 10  [ **IL10** ]  binding to PMN ran parallel to the kinetics of the inhibitory effect of IL 10  [ **IL10** ]  on the oxidative burst, suggesting a key role of IL 10R  [ **IL10RA** ]  mobilization from specific granules to the membranes in optimal regulation of inflammatory responses.  Ref: 11290804 J Immunol, 2001

**Non-interactive relationship** (e.g. studied together, co-existance, homology, etc.)

- The quaternary structures and the ability of IL 22 and IL 10  [ **IL10** ]  to engage soluble s IL 10R1  [ **IL10RA** ] , IL 22R, IL 10R2 receptor chains were analyzed using size exclusion chromatography and surface plasmon resonance techniques.  Ref: 12513909 J Interferon Cytokine Res, 2002
- In addition, exogenous IL 10  [ **IL10** ]  is highly potent in down regulating LPS induced IL 1beta and IL 10  [ **IL10** ]  mRNA, and, at a higher dose, IL 10R1  [ **IL10RA** ]  mRNA in untreated and LPS treated cultures, suggesting that IL 10  [ **IL10** ]  autoregulates its expression and inhibits that of IL 1beta at the transcriptional level.  Ref: 12405978 Eur J Neurosci, 2002
- IL 10R  [ **IL10RA** ]  expression did not correlate with plasma IL 10  [ **IL10** ]  levels or disease activity.  Ref: 12673894 Clin Exp Rheumatol< MedlineTA>Clin Exp Rheumatol, 2002
- Factor dependent cell progenitor promyeloid cells expressed functional IL 10Rs, as assessed by precipitation of a 110 kDa protein with an Ab to the IL 10R  [ **IL10RA** ]  and by the ability of IL 10  [ **IL10** ]  to activate Jak1 and Tyk2 and to phosphorylate tyrosine 705 on Stat 3.  Ref: 11591769 J Immunol, 2001
- Therefore, we have examined the expression of the anti inflammatory cytokines, interleukin 10  [ **IL10** ]  IL 10  [ **IL10** ]  and IL 4, and their specific receptors, IL 10R  [ **IL10RA** ]  and IL 4R, in postmortem human brain tissue obtained from MS patients.  Ref: 11921201 Glia, 2002
- During relapse, IL 10  [ **IL10** ] , IL 10R1  [ **IL10RA** ] , ICAM 1, VCAM 1 mRNA levels and neurological scores show positive correlations.  Ref: 12620647 J Neuroimmunol, 2003
- These findings suggest that Mphis, following thermal injury, display resistance to suppression by IL 10  [ **IL10** ]  due in part to downregulation of IL 10R  [ **IL10RA** ]  expression.  Ref: 11546654 Am J Physiol Cell Physiol, 2001
- Immune suppression was mediated independently of IL 10  [ **IL10** ]  and TGF beta production, as neutralizing antibodies for IL 10  [ **IL10** ] , IL 10R  [ **IL10RA** ] , and TGF beta were unable to revert suppression, and IL 10  [ **IL10** ]  deficient CD4 T cells were able to mediate in vitro and in vivo suppression.  Ref: 12609834 Blood, 2003
- Interleukin 10  [ **IL10** ]  receptor IL 10R  [ **IL10RA** ]  expression in human, dental pulp, fibroblast cultures was investigated by using the reverse transcriptase polymerase chain reaction RT PCR technique.  Ref: 12540220 J Endod, 2003
- The high levels of IL 10  [ **IL10** ]  and IL 10R  [ **IL10RA** ]  in foetal cartilage, an active growing tissue, suggest that IL 10  [ **IL10** ]  may play a role in controlling chondrocyte metabolism under physiological conditions.  Ref: 11332442 Clin Exp Rheumatol< MedlineTA>Clin Exp Rheumatol, 2003
- PURPOSE To investigate the mRNA expression of the receptors for tumour necrosis factor alpha TNFRp55, TNFRp75, interferon gamma IFN gamma R alpha, IFN gamma R beta, interleukin 10  [ **IL10** ]  IL 10R  [ **IL10RA** ] , CRFB4 and transforming growth factor beta TGF beta RII on human retinal pigment epithelium RPE cells and to modulate this expression with the pro inflammatory cytokines TNF alpha and IFN gamma as stimulators.  Ref: 11450494 Graefes Arch Clin Exp Ophthalmol, 2001
- Since neutrophils acquisition of responsiveness to IL 10  [ **IL10** ]  required de novo protein synthesis, we assessed whether expression of IL 10R1  [ **IL10RA** ]  or IL 10R2 was modulated in cultured neutrophils.  Ref: 11490020 J Immunol, 2001
- In established Leishmania donovani visceral infection in normal mice, anti interleukin IL 10  [ **IL10** ]  receptor IL 10R  [ **IL10RA** ]  monoclonal antibody MAb treatment induced intracellular parasite killing within liver macrophages.  Ref: 12870129 J Infect Dis, 2003
- IL 10  [ **IL10** ]  induced SOCS1 may directly inhibit IL 10  [ **IL10** ]  IFN gamma signaling, while inhibition of other proinflammatory cytokine responses may use additional IL 10R1  [ **IL10RA** ]  mediated mechanisms.  Ref: 12538698 J Immunol, 2003
- Semiautomated and simultaneous analysis of the interleukin 10  [ **IL10** ]  gene microsatellites IL 10G and IL 10R  [ **IL10RA** ]  by fluorescence based polymerase chain reaction reveals significant differences in allele distributions between Caucasians Germany and Africans Gabon.  Ref: 11566635 Eur Cytokine Netw< MedlineTA>Eur Cytokine Netw, 2003
- The significance of IL 10  [ **IL10** ]  and IL 10R  [ **IL10RA** ]  expression in renal cell carcinoma RCC has not been well characterized.  Ref: 11894117 Int J Oncol, 2002
- IL 10  [ **IL10** ]  s deactivating effects regulate outcome in experimental visceral leishmaniasis, and IL 10R  [ **IL10RA** ]  blockade represents a potential immuno and or immunochemotherapeutic approach in this infection.  Ref: 12379707 Infect Immun, 2002
- Collectively, our data emphasize the central role of IL 10R1  [ **IL10RA** ]  expression in regulating cell responsiveness to IL 10  [ **IL10** ] .  Ref: 12356680 Int Immunol, 2002
- To investigate the role of other loci in the susceptibility to RA, we have performed an analysis of several polymorphisms in genes of immune related function IL 10  [ **IL10** ]  108 1 92 promoter single nucleotide polymorphisms SNPs, IL 10G and IL 10R  [ **IL10RA** ]  microsatellites, IL 6 622 promoter SNP, FcgammaRIIIA Val Phe 158 polymorphism, IL 1 receptor antagonist VNTR, and the IKBL 738 T C mutation.  Ref: 12618859 Genes Immun, 2003
- In a rat model of chronic relapsing EAE, expression levels of IL 10  [ **IL10** ]  and its receptor IL 10R1  [ **IL10RA** ] , ICAM 1 and VCAM 1 mRNA in the spinal cord are markedly increased.  Ref: 12620647 J Neuroimmunol, 2003
- Using this method polymorphisms at the IL 10G and IL 10R  [ **IL10RA** ]  loci of the 5 flanking region of the IL 10  [ **IL10** ]  gene can be identified simultaneously.  Ref: 11566635 Eur Cytokine Netw< MedlineTA>Eur Cytokine Netw, 2003
- These data demonstrate that IL 10R1  [ **IL10RA** ]  expression plays a critical role in determining whether cells respond to IL 10  [ **IL10** ] .  Ref: 11739506 J Immunol, 2001
- In IL 10R1  [ **IL10RA** ]  G330R expressing monocytes, the inhibitory effect of IL 10  [ **IL10** ]  on TNF alpha production was diminished, indicating that this variant may be a loss of function allele.  Ref: 12759436 J Immunol, 2003
- Blocking of murine IL 10  [ **IL10** ]  receptor IL 10R  [ **IL10RA** ]  in vivo shortened the survival time of highly susceptible T. congolense infected BALB c mice.  Ref: 12594839 Eur J Immunol, 2003
- As judged by IL 10R  [ **IL10RA** ]  blockade, endogenous IL 10  [ **IL10** ]  primarily regulates killing in L. donovani infection by suppressing production of and responses to the Th1 cell type cytokines, IL 12, and IFN gamma.  Ref: 12870129 J Infect Dis, 2003
- However, because anti IL 10R  [ **IL10RA** ]  also released IFN gamma independent effects, IL 10  [ **IL10** ]  appears to act more broadly and suppresses multiple antileishmanial mechanisms.  Ref: 12870129 J Infect Dis, 2003
- Modulation of cell surface IL 10R1  [ **IL10RA** ]  density might be an important mechanism for determining whether IL 10  [ **IL10** ]  leads to immunostimulation or immunosuppression in vivo.  Ref: 11739506 J Immunol, 2001
- In contrast, surface IL 10R1  [ **IL10RA** ]  and IL 10  [ **IL10** ]  binding activity diminished in mature DCs.  Ref: 11254683 J Immunol, 2001
- The highest levels of IL 10  [ **IL10** ]  protein and IL 10R  [ **IL10RA** ]  were found in foetal cartilage.  Ref: 11332442 Clin Exp Rheumatol< MedlineTA>Clin Exp Rheumatol, 2001
- OBJECTIVE We aimed to determine the expression of the interleukin 10  [ **IL10** ]  receptor IL10R  [ **IL10RA** ]  on circulating leukocytes in SLE and rheumatoid arthritis, and correlate this with plasma IL 10  [ **IL10** ]  levels and disease activity.  Ref: 12673894 Clin Exp Rheumatol< MedlineTA>Clin Exp Rheumatol, 2001
- Endogenous IL 10  [ **IL10** ]  and IL 10R  [ **IL10RA** ]  can be relevant targets for the manipulation of DC functions.  Ref: 11254683 J Immunol, 2001
- We examined IL 10  [ **IL10** ]  and IL 10  [ **IL10** ]  receptor IL 10R  [ **IL10RA** ]  gene expression in 44 xenografts of human non small cell lung cancer NSCLC by reverse transcription polymerase chain reaction RT PCR assay with human specific primers.  Ref: 11351253 Int J Oncol, 2001
- To develop a rationale for IL 10  [ **IL10** ]  inhibition as treatment in visceral infection, Th1 cytokine driven responses were characterized in Leishmania donovani infected BALB c mice in which IL 10  [ **IL10** ]  was absent or overexpressed or its receptor IL 10R  [ **IL10RA** ]  was blockaded.  Ref: 12379707 Infect Immun, 2002
- Furthermore, intravenous LPS upregulated expression of IL 10  [ **IL10** ]  receptor IL 10R1  [ **IL10RA** ]  mRNA in the liver.  Ref: 12010759 Am J Physiol Regul Integr Comp Physiol, 2002
- Structural analysis of the S138G variant revealed that the substitution of S138G may interfere with binding of IL 10  [ **IL10** ]  to IL 10R1  [ **IL10RA** ] .  Ref: 12759436 J Immunol, 2003
- In the present study we describe the expression of IL 10  [ **IL10** ]  and the IL 10  [ **IL10** ]  receptor IL 10R1  [ **IL10RA** ]  in primary cocultures of rat microglial and astroglial cells.  Ref: 12405978 Eur J Neurosci, 2002
- Expression of the inhibitory cytokine IL 10  [ **IL10** ]  and the IL 10R  [ **IL10RA** ]  are sustained after induction of LPS tolerance.  Ref: 11500393 Infect Immun, 2001
- This shows concomitant induction of IL 10R  [ **IL10RA** ]  but not CRF2 4 to that of IL 10  [ **IL10** ]  by activated HSC in vitro and in vivo and associated acquisition of the responsiveness to IL 10  [ **IL10** ] , entailing complex effects on HSC.  Ref: 12016123 Am J Physiol Gastrointest Liver Physiol, 2002
- Our results demonstrate that the capacity of neutrophils to respond to IL 10  [ **IL10** ] , as assessed by Stat3 tyrosine phosphorylation, SOCS 3 expression, and modulation of cytokine production, is very dependent on the level of expression of IL 10R1  [ **IL10RA** ] .  Ref: 11490020 J Immunol, 2001
- A single mutation Ile87Ala in the same CD loop region of the Epstein Barr virus IL 10  [ **IL10** ]  ebvIL 10 also reduces IL 10R1  [ **IL10RA** ]  binding affinity, suggesting that ebvIL 10 and 9D7Fab use similar allosteric mechanisms to modulate IL 10R1  [ **IL10RA** ]  affinity and biological activity.  Ref: 12121653 Structure Camb , 2002
- OBJECTIVE To evaluate the expression of interleukin 10  [ **IL10** ]  IL 10  [ **IL10** ]  and interleukin 10  [ **IL10** ]  receptor IL 10R  [ **IL10RA** ]  on chondrocytes from healthy, osteoarthritic, and foetal cartilage from human subjects.  Ref: 11332442 Clin Exp Rheumatol< MedlineTA>Clin Exp Rheumatol, 2002
- We have shown that there is significant TNF alpha activity in patients with IPF, despite the presence of significant amounts of IL 10  [ **IL10** ]  and Il 10R  [ **IL10RA** ] .  Ref: 11282543 Eur Cytokine Netw, 2001
- Purification, crystallization and preliminary X ray diffraction of a complex between IL 10  [ **IL10** ]  and soluble IL 10R1  [ **IL10RA** ] .  Ref: 11717514 Acta Crystallogr D Biol Crystallogr, 2001
- Crystal structure of the IL 10  [ **IL10** ]  IL 10R1  [ **IL10RA** ]  complex reveals a shared receptor binding site.  Ref: 11485736 Immunity, 2001
- IL 10  [ **IL10** ]  induced SOCS is associated with the inhibition of IFN gamma signaling in various cell types, and this inhibition is independent of C terminal serine residues of the IL 10R  [ **IL10RA** ] , previously shown to be required for other anti inflammatory responses.  Ref: 12538698 J Immunol, 2003
- Because IL 10  [ **IL10** ]  is a potent inhibitor of IL 12 synthesis from activated human mononuclear cells, we assessed the inhibitory potential of this cytokine using soluble IL 10R  [ **IL10RA** ] .  Ref: 11390447 J Immunol, 2001
- We studied three SNPs  **IL10**  108 24, and 597 and two microsatellites IL10R  [ **IL10RA** ]  and IL10G lying within the promoter region of  **IL10**  for association with susceptibility to and clinical manifestations of ankylosing spondylitis AS, a common form of spondyloarthritis.  Ref: 12595905 Genes Immun, 2003
